# Changes in Family Caregiver Roles and Interactions During the COVID-19 Pandemic

**DOI:** 10.1093/geroni/igaa057.3427

**Published:** 2020-12-16

**Authors:** Rajean Moone, Elizabeth Lightfoot Kamal Abdi Suleiman, Courtney Kutzler, Jacob Otis, Kenneth Turck, Heejung Yun

**Affiliations:** 1 University of Minnesota, Woodbury, Minnesota, United States; 2 University of Pennsylvania, St. Paul, Minnesota, United States; 3 University of Minnesota, St. Paul, Minnesota, United States

## Abstract

Family caregivers provide the majority of support for older adults and people with disabilities in the U.S. The onset of the COVID-19 pandemic forced radical changes in duties and relationships between family caregivers and care recipients. These changes can be attributed to fears of virus transmission as well as federal, state and local government mitigation strategies resulting in social distancing and quarantining limiting caregiving interactions. This qualitative investigation conducted 55 Zoom interviews over summer 2020 with family caregivers to explore their changing roles and duties during the pandemic. Researchers utilized a semi-structured interview guide to explore caregiver experiences with COVID-19. The average age of the caregiver participants was 59 and the average age of the care recipients for whom they provided care was 74. All participants provided unpaid care for family members. Interviews were conducted in English (n=40), Spanish (n=5), Somali (n=5) and Korean (n=5). Care recipients resided in a facility (nursing home, memory care, ICF-DD, or other assisted living) (70%) with the caregiver (20%), and in a separate independent setting (10%). Data from each interview were coded into themes by two researchers. Themes that emerged from the analyses included concerns about care recipient mental and physical health deterioration, lack of communication from formal providers, change in relationships with other family members, and future concerns. Implications for additional research and practice are included.

